# Trends and determinants of nurses’ mental health following the COVID-19 pandemic in China: a longitudinal, comparative study over a two-year period

**DOI:** 10.3389/fpsyt.2024.1480969

**Published:** 2024-11-07

**Authors:** Yan Liu, Yifei Lin, Wenyao Cui, Xianlin Gu, Youlin Long, Wenjie Liu, Ga Liao, Liang Du, Jin Huang

**Affiliations:** ^1^ Department of Neurosurgery, West China Hospital, Sichuan University/West China School of Nursing, Sichuan University, Chengdu, China; ^2^ West China Hospital, Sichuan University, Chengdu, China; ^3^ Health Management Center, General Practice Medical Center, West China Hospital, Sichuan University, Chengdu, China; ^4^ Medical Device Regulatory Research and Evaluation Centre, West China Hospital, Sichuan University, Chengdu, China; ^5^ Department of Rehabilitation Medicine, West China Hospital, Sichuan University, Chengdu, China; ^6^ Chinese Evidence-Based Medicine Center, West China Hospital, Sichuan University, Chengdu, China; ^7^ State Key Laboratory of Oral Diseases, National Clinical Research Center for Oral Diseases, West China Hospital of Stomatology, Sichuan University, Chengdu, China; ^8^ West China Medical Publishers, West China Hospital, Sichuan University, Chengdu, China; ^9^ Health Management Center, General Practice Medical Center, Medical Device Regulatory Research and Evaluation Center, Chinese Evidence-Based Medicine Center, West China Hospital, Sichuan University, Chengdu, China

**Keywords:** COVID-19, pandemics, nurses, psychological distress, post-traumatic stress disorders, longitudinal study

## Abstract

**Introduction:**

The COVID-19 pandemic has placed nurses at the forefront of healthcare, exposing them to various mental health challenges such as depression, anxiety, and post-traumatic stress disorder (PTSD). However the long-term effects and risk factors of the COVID-19 pandemic on nurses’ mental health are unknown. The objective of our study is to investigate the enduring impact of the COVID-19 pandemic on the psychological distress and PTSD of nurses, while also identifying the factors that influence these outcomes

**Methods:**

To investigate this question, we conducted a self-reported questionnaire survey of 8785 registered nurses recruited using snowball sampling methodology from 22 provinces in China, at five time points (T0-T4). At all times, we took measures of general health, while at the last four times we also measured PTSD. And we used logistic regression analysis to explore their impacts.

**Results:**

The incidence of whole levels psychological distress among nurses was 27.7% at T0, peaking at 57.6% at T4. For severe psychological distress, it began at 5.5% at T0 and rose to 9.5% at T4. PTSD rates among nurses were recorded at 7.8% in T1, reaching a maximum of 14.7% in T4. They all got progressively worse in the volatility. In all times, coping strategies are independent factors for various levels of psychological distress and PTSD, while social support is a key factor for severe psychological distress and PTSD.

**Conclusions:**

Our findings indicated a gradual deterioration in the mental health of nurses following the COVID-19 outbreak. In all instances, coping strategies exhibit an independent correlation with all grades of psychological distress and post-traumatic stress disorder (PTSD), whereas social support emerges as an independent protective factor mitigating the risk of severe psychological distress and PTSD.

## 1 Introduction

The COVID-19 pandemic, which emerged in early 2020, has had a profound and far-reaching impact on global mental health, prompting widespread research attention (1, 2). The research on the potential hazards of the COVID-19 pandemic to Asian nurses mainly focuses on personalized mental health care (3). Among these, healthcare workers, particularly nurses, have emerged as a vulnerable group, facing unique challenges that have threatened their mental well-being (4, 5).

Recent studies have highlighted the high prevalence of mental health issues among nurses during the COVID-19 pandemic. For instance, in Saudi Arabia, rates of generalized anxiety disorder and depressive disorder among healthcare providers reached 51.4% and 55.2%, respectively (6). This rate is the highest we have found so far. Similarly, in various countries, hospital nurses reported substantial anxiety, depressive symptoms, and sleep disturbances (7). Meta-analyses and empirical studies have further emphasized the elevated risk of PTSD among nurses, with incidence rates surpassing those of doctors and COVID-19 patients (8, 9). Healthcare professionals, particularly women and nurses, have emerged as vulnerable populations experiencing heightened mental health challenges (8, 10, 11).

Several studies have consistently reported substantial rates of depression and anxiety among nurses, specifically noting incidences of 34.3% for depression and 18.1% for anxiety (12). These findings echo our previous research, which revealed a 28% incidence of psychological distress among nurses during the pandemic (13), highlighting the widespread impact on the mental well-being of this essential workforce.

However, despite these concerning trends, the dynamics and longitudinal effects of COVID-19 on the mental health of nurses remain understudied, particularly in the Chinese context. Research has suggested regional variations in the severity and prevalence of mental health problems among nurses, with some studies indicating improvements over time while others highlight worsening symptoms (14, 15). A study from China found varying incidence rates of depression, anxiety, insomnia and PTSD among nurses involved in supporting Wuhan’s anti-epidemic efforts, with higher rates ovserved one year later compared to those who were not involved (16). A longitudinal study conducted in Australia revealed that depression and PTSD symptoms worsened over time among first-line perioperative healthcare staff, suggesting that their mental health continued to be affected even as the pandemic subsided (15). Similar studies conducted in other regions have also indicated short- and medium-term mental health effects of COVID-19 on nurses (17).

During the battle against the COVID-19 pandemic, front-line medical staffs have faced significant psychological pressure, but research on the dynamic monitoring of mental health changes following COVID-19 remains limited (11). Although the pandemic has somewhat abated, its pasychological impact persists (15). The long-term psychological consequences of the COVID-19 pandemic maysurpass its economic ramifications, posing a substantial challenge for healthcare organizations (7). Therefore, it is crucial to provide sustained attention to the mental health of nurses in the aftermath of the COVID-19 outbreak to ensure the stability of the healthcare system. Yet, there is currently a lack of long-term research on the mental health of Chinese nurses following the COVID-19 outbreak. This highlights the need for comprehensive, longitudinal studies to better understand the mental health trajectories of nurses in the aftermath of the pandemic.

Addressing the gap in knowledge, this study aims to conduct a comprehensive longitudinal investigation into the mental health of Chinese nurses. Our focus is on examining the trends in general health status and PTSD, as well as the factors influencing these outcomes, following the initial wave of the COVID-19 pandemic. By comparing mental health states during the subsequent wave with those observed in the initial wave, we seek to identify any changes or patterns that may inform intervention strategies to support the mental wellbeing of Chinese nurses in the long term. The results of this study are expected to provide vital insights for healthcare organizations and policymakers, contributing to the development of effective mental health support systems for nurses in the post-pandemic era.

## 2 Methods

### 2.1 Study design and participants

We conducted a cross-sectional study to investigate the mental health of Chinese nurses during the first big wave of the COVID-19 pandemic, between Feb 11^th^ and 18^th^, 2020. The initial study found that the incidence of psychological distress among nurses, assessed using the General Health Questionnaire (GHQ-28) with a score >5, was 28% (13). Subsequently, we conducted four longitudinal follow-up surveys after the first wave of the pandemic, with a follow-up during the second big wave.

The calculation of the sample size adheres to the Kendall sample estimation methodology, formulated as N = [Max(number of items) * (10 to 20)] * [1 + (10% to 20%)] (18). In this study, the GHQ-28 scale, consisting of 28 items, was employed. To account for potential attrition and invalid questionnaires, while maintaining the representativeness of the study population as comprehensively as possible, an additional 20% adjustment was applied. Consequently, the final sample size was determined using the formula N = [Max(number of items) * 20] * (1.20). Upon substituting the number of items, the minimum required sample size was calculated to be 672.

The study involved five time points: T0, T1, T2, T3, and T4. Questionnaires were distributed using the “SO JUMP platform”, a professional online questionnaire platform, to all invited nurses. “SO JUMP platform” employs SSL (Secure Sockets Layer) or other encryption technologies to safeguard the security of data transmission, ensuring that user information is not intercepted or tampered with by unauthorized third parties. Only authorized personnel are granted access to view the relevant data. During the completion process, we did not ask participants to provide any information that involves privacy (such as name, contact details, etc.). When publishing the questionnaire on “SO JUMP platform”, we included a consent form at the beginning and set a requirement for completion before successful submission, thereby ensuring the integrity of the collected data. The questionnaires were administered at the following time points T0(between Feb 11^th^ and 18^th^, 2020), T1(between Apr 8^th^ and 14^th^, 2020), T2(between Jul 8^th^ and 14^th^, 2020), T3(between Nov 8^th^ and 14^th^, 2020), and T4(between May 8^th^ and 14^th^, 2022). Time point 1 coincieded with the first wave of the COVID-19 pandemic, while time point 2 occurred after the 76-day lockdown on Wuhan was lifted on April 8, 2020 (19). At the time point 2, most front-line nurses, particularly those who supported the epidemic response in Wuhan, had returned to normal work for more than one month. Time point 3 represented approximately six months since the first wave of the pandemic ended in China, and time point 4 corresponded to the second big wave of the COVID-19 pandemic.

### 2.2 Survey promotion and distribution

At T0 (between Feb 11^th^ and 18^th^ 2020), we investigated the mental health and influencing factors of Chinese nurses under the COVID-19 pandemic. Subsequently, we conducted four longitudinal follow-up questionnaires using the same questionnaire delivery method with the same target population at T1 (between Apr 8^th^ and 14^th^, 2020), T2 (between Jul 8^th^ and 14^th^, 2020), T3 (between Nov 8^th^ and 14^th^, 2020), and T4(between May 8^th^ and 14^th^, 2022). To ensure consistency, the questionnaires were strictly sent to every subject who received the questionnaire at T0, and participants were instructed to forward the questionnaire to all individuals they had initially sent it to at T0.

### 2.3 Questionnaire

The questionnaire covered six areas: participant demographics, work experience during the pandemic, social support, stress coping strategies, psychological distress and post-traumatic stress disorder.

Demographic data, including age, gender, education background, professional title, years of service, job position, marital status, exposure level, living with a child, and living alone, were collected. High exposure level was defined as first-line nurses directly involved in treating COVID-19 patients, while low exposure level referred to second-line nurses who did not directly treat COVID-19 patients.

Psychological distress was assessed using the General Health Questionnaire-28 (GHQ-28), a 28-item questionnaire that measured depression, anxiety, social impairment, and somatic symptoms. GHQ-28 scores were calculated using a bisection scoring procedure (0-0-1-1), with total scores ranging from 0 to 28. Score above 5 indicated psychological distress,while scores above 11 indicated severe psychological distress (20, 21).

To measure the presence of PTSD symptoms, we employed the Post-Traumatic Stress Disorder Self-Rating Scale (PTSD-ss), developed according to the diagnostic criteria of PTSD outlined in the Diagnostic and Statistical Manual of Mental Disorders, Fourth Edition (DSM-IV) and the Chinese Classification of Mental Disorders. The scale consists of 24 items, each rated on a five-point Likert scale from 1 (‘not at all’) to 5 (‘extremely severe’). A cut-off total score of 50 was used to identify probable PTSD (22).

The social support survey section evaluates the adequacy of support received in 11 areas, including “support from relatives and friends,” “society’s gratitude and appreciation,” “hospital’s protective facilities and temporary accommodation arrangements,” “provision of insurance and compensation if infected at the workplace,” “teamwork and collaboration among colleagues,” “gratitude from patients and their relatives,” “clear guidelines for new work arrangements and infection control,” “acceptance of feedback from frontline staff by administrative-level personnel,” “employee psychological counseling organized by superior management departments or hospitals,” “opportunity to voice opinions through labor unions or mass media,” and “others.” All 11 questions are answered with either “adequate” or “inadequate,” with 1 point awarded for “adequate” and no points for “inadequate,” resulting in a maximum possible score of 11 points.

The stress coping strategies questionnaire utilized in this study comprises a total of 13 items, designed to inquire about the frequency with which nursing personnel employed 13 distinct coping strategies during the outbreak of the pandemic. All items employ a 4-point rating scale, denoted by numbers 1 to 4, where (1=Never, 2=Occasionally, 3=Frequently, 4=Always). The 13 coping strategies are as follows: “Talking with colleagues, friends, or relatives,” “Losing temper and shifting anger onto others,” “Seeking professional consultation for psychological issues,” “Seeking support from religious beliefs,” “Using alcohol or drugs to alleviate negative emotions,” “Redirecting attention away from work towards other relaxing activities,” “Expressing opinions to superiors or administrative staff,” “Venting emotions through social media,” “Actively learning about progress and seeking solutions,” “Adopting a positive attitude towards adversity,” “Giving up on or avoiding difficult problems,” “Blaming oneself,” and “Blaming others.”

### 2.4 Statistical analysis

To achieve the objectives of the study, a comprehensive statistical analysis plan was designed. Statistical analysis was performed using R software. The primary analyses focused on:

#### 2.4.1 Descriptive statistics

All collected questionnaires were sorted and the data were entered into Microsoft Excel for analysis. Data were summarized using means and standard deviations or medians and interquartile ranges for continuous variables, while categorical variables were summarized using frequencies. A significance level (alpha) of 0.05 was set, and all tests were two-tailed.

#### 2.4.2 To compare the incidence of psychological distress, severe psychological distress, and PTSD between first-line and second-line nurses, as well as between different time points

Chi-square analyses, Wilcoxon rank-sum tests, and two-tailed independent samples T-tests were employed with Bonferroni correction to control for type I error. Specifically, comparisons of the incidence of psychological distress (total GHQ scores above 5), severe psychological distress (total GHQ scores of 11 or above), and PTSD (total PTSD-ss scores above 50) between T0, T1, T2, T3, and T4 in all nurses, first-line nurses, and second-line nurses were adjusted using Bonferroni correction. The incidence of psychological distress and severe psychological distress between T0, T1, T2, T3, and T4 in all nurses, first-line nurses and second-line nurses was compared 10 times, so alpha was corrected to 0.005. The incidence of PTSD between T0, T1, T2, T3 and T4 in all nurses, first-line nurses, and second-line nurses was compared 10 times, resulting in a corrected alpha of 0.005. Similarly, the incidence of PTSD between T1, T2, T3, and T4 in all nurses, first-line nurses, and second-line nurses was compared six times, resulting in a corrected alpha of 0.0083 (23).

#### 2.4.3 Regression analyses

To explore factors associated with psychological distress, severe psychological distress, and PTSD. Multivariable logistic regression analyses were conducted, examining the influence of demographic variables, participation in treating COVID-19 patients, social support, and coping strategies. Missing data were imputed using the sample mean for the respective variable.

### 2.5 Ethical approval

The study was approved by the Biomedical Research Ethics Committee, West China Hospital of Sichuan University. The survey was conducted anonymously, and the personal information was not disclosed except for demographic data. Informed consent was obtained from participants, who were provided with an initial section in the questionnaire outlining the purpose and procedures of the study. Participants could choose to proceed with the questionnaire by clicking “I agree”. Participants had the right to withdraw from the study at any time without consequences.

## 3 Results

### 3.1 Participant characteristics

A total of 8785 participants contributed data to at least one of the five web surveys, with 7385 (84.1%) female and 1400 (15.9%) male participants. Valid questionnaires were collected from 1364 participants at T0, 1081 at T1, 1221 at T2, 1839 at T3, and 3280 at T4 (**Table 1**). The number of valid questionnaires collected varied across time points, reflecting the longitudinal nature of the study.

**Table 1:** Demographic characteristics of the participants.

	T0 (n=1364)	T1 (n=1081)	T2 (n=1221)	T3 (n=1839)	T4 (n=3280)	H/χ2	P-Value
**Age, median (IQR), years**	30 (27-34)	31 (27-35)	30 (27-35)	30 (26-34)	31 (27-36)	H=36.139	<0.001
**Gender (Female/Male), n**	1072/292	856/225	1014/207	1615/224	2828/452	χ2 = 81.363	<0.001
**Education background, n (%)**						H=3362.458	<0.001
Phd	6	7	4	2	2		
Master	40	45	31	18	91		
Bachelor	1032	796	928	1196	2283		
College degree and others	286	233	258	623	904		
**Professional Title, n (%)**						H=4753.371	<0.001
Advanced	75	70	67	107	178		
Medium-garde	386	308	342	506	924		
Primary	903	703	812	1226	2178		
**Years of service, median (IQR), years**	8 (4-12)	8 (5-12)	8 (5-12)	8 (4-12)	9 (5-13)	H=35.374	<0.001
**Manager, n (%)**	268/1096	225/856	258/963	410/1429	688/2592	χ2 = 3.380	0.496
**Marital status, n (%)**						χ2 = 15.752	0.046
Married	868	711	853	1201	2152		
Unmarried	463	352	347	591	1065		
Divorced	33	18	21	47	63		
**Living with a child, n (%)**	799/565	633/448	752/469	1082/757	1941/1339	χ2 = 3.373	0.497
**Lives alone, n (%)**	447/917	324/757	370/851	536/1303	892/2388	χ2 = 15.803	0.003

### 3.2 Prevalence of psychological distress and PTSD among all nurses

**Figure 1:**
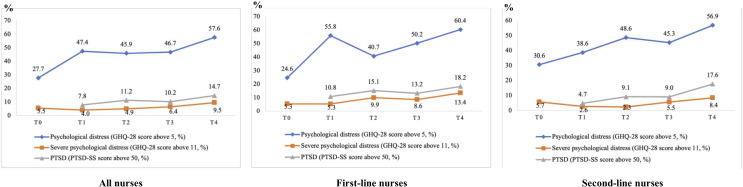
The incidence of psychological distress and PTSD among nurses.

The incidence rates of psychological distress and PTSD among all nurses, first-line nurses, and second-line nurses are presented in **Table 2** and **Figure 1**. The results revealed significant variations in the prevalence of psychological distress, severe psychological distress, and PTSD among nurses over time and between first-line and second-line nurses. The prevalence rates were reported for each time point, and significant differences were identified through statistical comparisons. Among all nurses, the rates of psychological distress and severe psychological distress were 27.7% and 5.5% at T0, 47.4% and 4.0% at T1, 45.9% and 5.0% at T2, 46.7% and 6.4% at T3, and 57.6% and 9.5% at T4, respectively. The incidence of PTSD among all nurses was 7.8% at T1, 11.2% at T2, 10.2 at T3, 14.7 at T4. First-line nurses exhibited rates of psychological distress and severe psychological distress of 24.7% and 5.3% at T0, 51.8% and 0.9% at T1, 53.9% and 5.4% at T2, 54.5% and 5.9% at T3, and 57.4% and 12.2% at T4, respectively. The incidence of PTSD among first-line nurses was 6.0% at T1, 11.8% at T2, 10.0% at T3, and 18.9% at T4. The rates of psychological distress and severe psychological distress among second-line nurses was 30.6% and 5.7% at T0, 42.8% and 7.1% at T1, 41.6% and 4.6% at T2, 43.5% and 6.6% at T3, and 57.7% and 8.8% at T4, respectively. The incidence of PTSD among second-line nurses were 9.6% at T1, 10.9% at T2, 10.3% at T3, and 13.5% at T4.

**Table 2:** Prevalence of psychological distress and PTSD among Chinese nurses during and after the COVID-19 pandemic.

	N(%)T0			N(%)T1			N(%)T2			N(%)T3			N(%)T4		
	All(n=1364)	First- Line(n=658)	Second-line(n=706)	All(n=1081)	First- line(n=548)	Second-line(n=533)	All(n=1221)	First- line(n=423)	Second-line(n=798)	All(n=1839)	First- line(n=538)	Second-line(n=1301)	All(n=3280)	First- line(n=714)	Second-line(n=2566)
**Psycological distress** (GHQ-28 score above 5)	378 (27.7)	162 (24.6)	216 (30.6)	512 (47.4)	306 (55.8)	206 (38.6)	560 (45.9)	172 (40.7)	388 (48.6)	859 (46.7)	270 (50.2)	589 (45.3)	1890 (57.6)	431 (60.4)	1459 (56.9)
**Severe psychological distress** (GHQ-28 score above 11)	75 (5.5)	35 (5.3)	40 (5.7)	43 (4.0)	29 (5.3)	14 (2.6)	60 (4.9)	42 (9.9)	18 (2.3)	118 (6.4)	46 (8.6)	72 (5.5)	312 (9.5)	96 (13.4)	216 (8.4)
**PTSD** (PTSD-ss score above 50)	\	\	\	84 (7.8)	59 (10.8)	25 (4.7)	137 (11.2)	64 (15.1)	73 (9.1)	188 (10.2)	71 (13.2)	117 (9.0)	482 (14.7)	130 (18.2)	452 (17.6)

### 3.3 Comparisons of psychological distress and PTSD across time points

**Figure 2:**
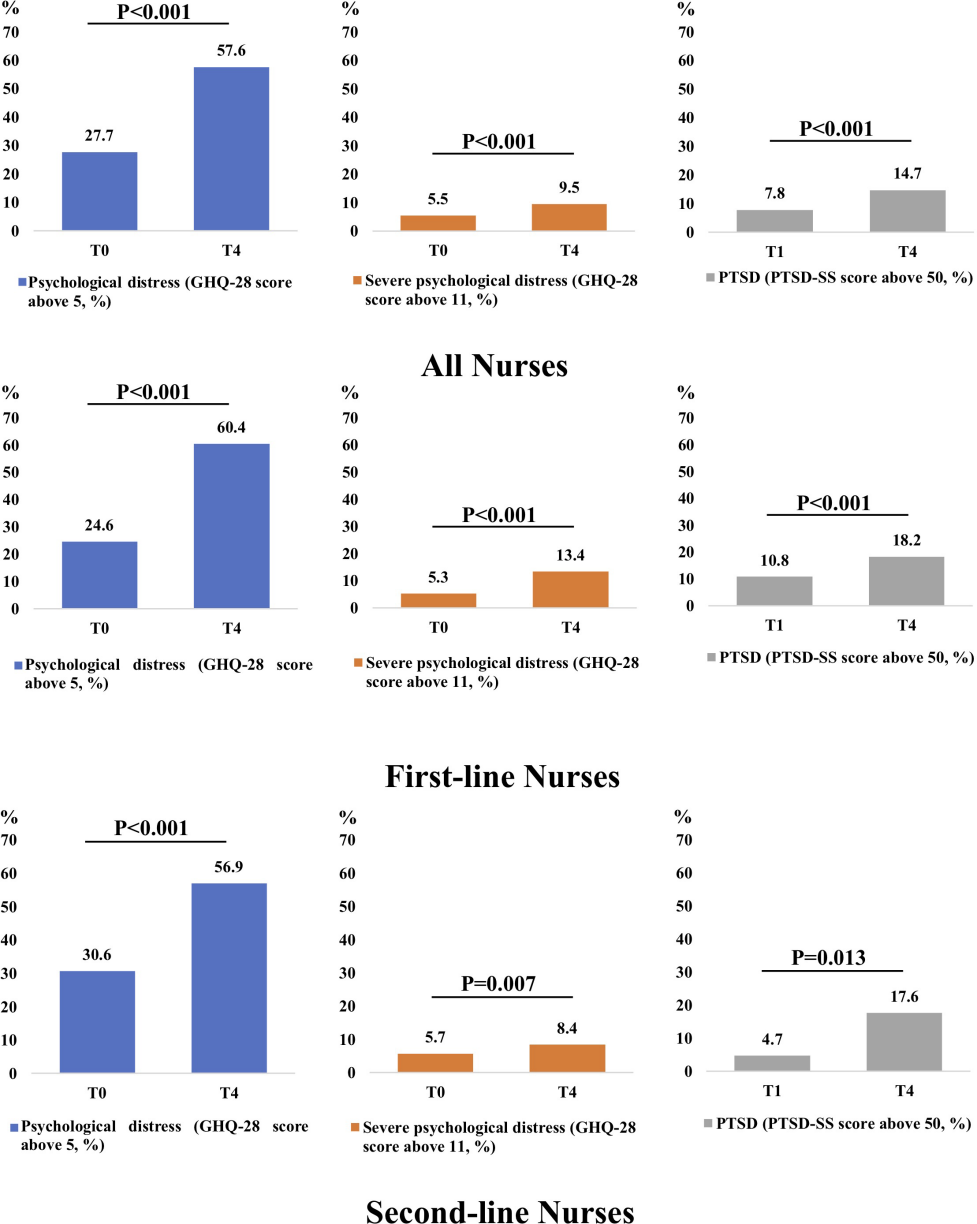
Comparison of the incidence of psychological distress between T0 and T4 and comparison of incidence of PTSD between T1 and T4.

Significant increases in the prevalence of psychological distress, severe psychological distress, and PTSD were observed from T0 (first wave of the pandemic) to T4 (second big wave of the pandemic). This pattern suggests a cumulative impact of the pandemic on nurses’ mental health. The prevalence of the psychological distress among all nurses was 27.7% at T0 and 57.6% at T4 (P<0.001). The prevalence of severe psychological distress among all nurses was 5.5% at T0 and 9.5% T4 (P<0.001). The prevalence of PTSD among all nurses was 7.8% at T1 and 14.7% at T4 (P<0.001). The prevalence of psychological distress among first-line nurses was 24.6% at T0 and 60.4% at T4 (P<0.001). The prevalence of the severe psychological distress among first-line nurses was 5.3% and 13.4% at T0 and T4 (P<0.001). The prevalence of PTSD among first-line nurses was 10.8% at T1 and 18.2% at T4 (P<0.001).The prevalence of psychological distress among second-line nurses was 30.6% at T0 and 56.9% at T4 (P<0.001). The prevalence of severe psychological distress among second-line nurses was 5.7% at T0 and 8.4% at T4 (P=0.007). The prevalence of PTSD among second-line nurses was 4.7% at T1 and 17.6% at T4 (P<0.001)s (**Figure 2**; **Supplementary Material 1**, **Supplementary Materials 2–3**).

### 3.4 Comparisons of psychological distress and PTSD between first-line nurses and second-line nurses

**Figure 3:**
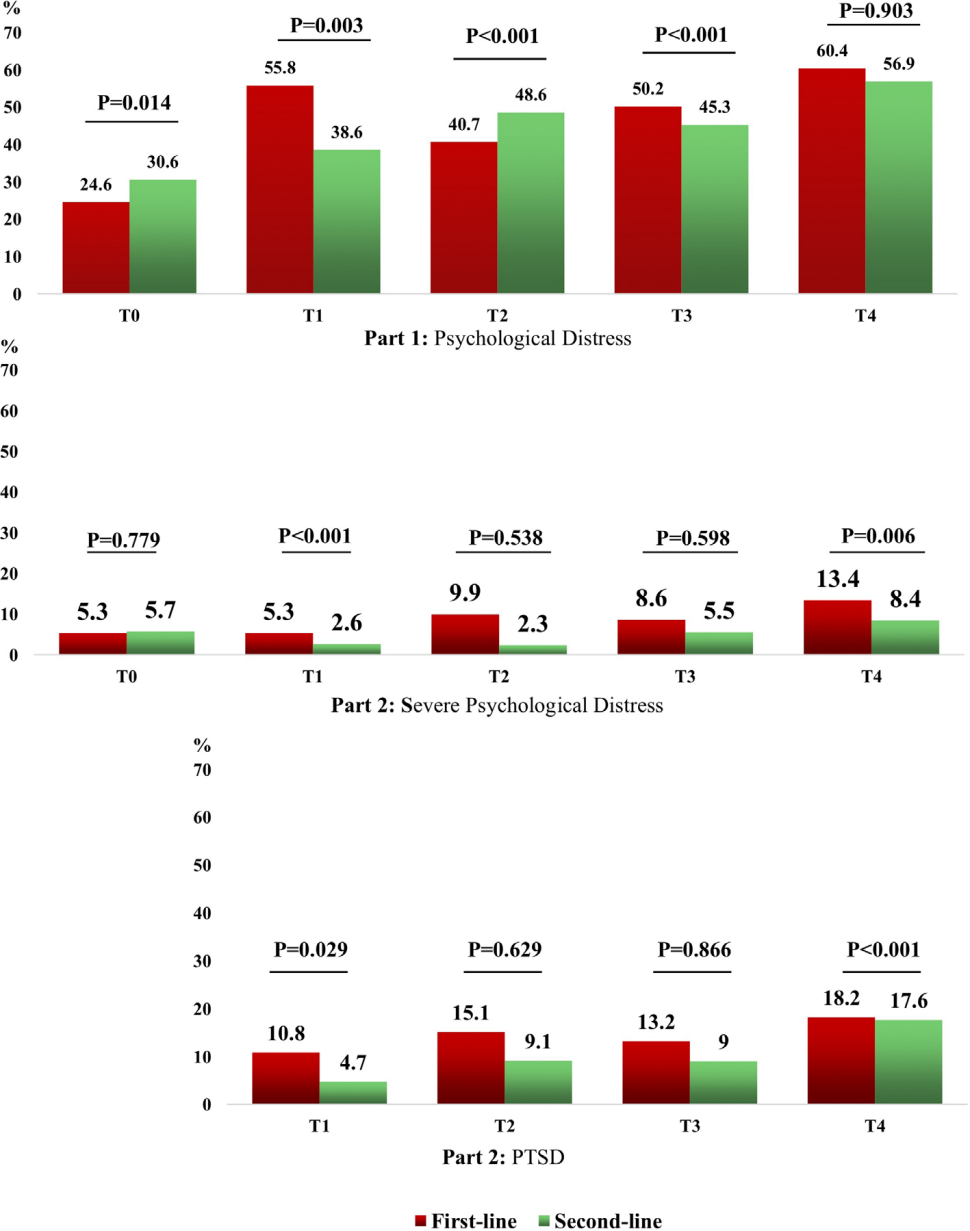
Comparison of the incidence of Psychological Problems between first-line and second-line nurses.

Statistically significant differences were found in the incidence of psychological distress and PTSD between first-line and second-line nurses at certain survey time points. These findings highlight the unique challenges faced by front-line nurses directly involved in treating COVID-19 patients. Statistically significant differences in the incidence of psychological distress and PTSD between first-line and second-line nurses were observed at certain survey time points. Significant differences in the incidence of psychological distress between first-line and second-line nurses were found at T0 (P=0.014), T1 (P=0.003), T2 (P<0.001) and T3 (P<0.001). Significant differences in the incidence of severe psychological distress between first-line and second-line nurses were observed at T1 (P<0.001) and T4 (P=0.006). Significant differences in the incidence of PTSD between first-line and second-line nurses were found at T1 (P=0.029) and T4 (P<0.001) (**Figure 3**; **Supplementary Material 1**, **Supplementary Materials 2–3**).

### 3.5 Risk factors associated with psychological distress

**Figure 4:**
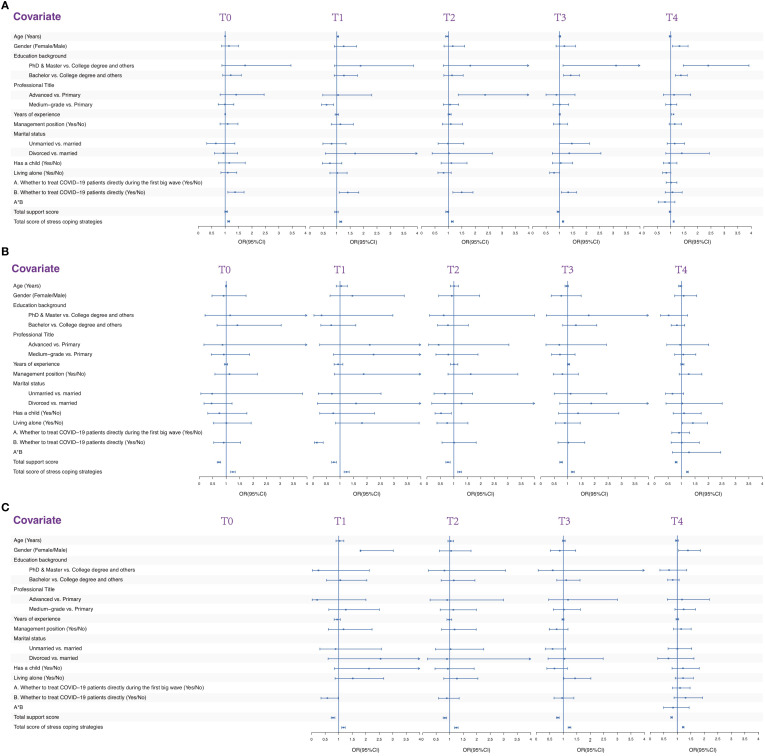
Multivariable logistic regression analysis of the factors associated with psychological problems. **(A)** Multivariable logistic regression analysis of the factors associated with psychological distress. **(B)** Multivariable logistic regression analysis of the factors associated with severe psychological distress. **(C)** Multivariable logistic regression analysis of the factors associated with PTSD.

Logistic regression analyses revealed that higher total score of stress coping strategies was associated with an increased risk of whole levels psychological distress, suggesting that some coping strategies may not be effective or may even exacerbate negative outcomes. The results of logistic regression analysis examining the factors associated with psychological distress are presented in **Figure 4** and **Table 3**. Directly treating COVID-19 patients was associated with a higher risk of psychological distress at T0 (OR, 1.370; 95% CI, 1.101 – 1.705), T1 (OR, 1.409; 95% CI, 1.091 – 1.819), T2 (OR, 1.495; 95% CI, 1.167 – 1.916), and T3 (OR, 1.324; 95% CI, 1.069 – 1.641). A higher total score of stress coping strategies was also associated with an increased risk of psychological distress at T0 (OR, 1.130; 95% CI, 1.094 – 1.167), T1 (OR, 1.148; 95% CI, 1.108 – 1.189), T2 (OR, 1.144; 95% CI, 1.107 – 1.182), T3 (OR, 1.131; 95% CI, 1.103 – 1.160) and T4 (OR, 1.109; 95%CI, 1.089 – 1.129).

**Table 3:** Multivariable logistic regression analysis of the factors associated with psychological distress.

	T0		T1		T2		T3		T4	
	OR(95%CI)	P Value	OR(95%CI)	P Value	OR(95%CI)	P Value	OR(95%CI)	P Value	OR(95%CI)	P Value
**Age (Years)**	0.999(0.997-1.002)	0.516	1.037(1.006-1.069)	0.019*	0.949(0.894-1.007)	0.085	1.014(0.984-.045)	0.358	0.977(0.943-1.012)	0.198
**Gender (Male/Female)**	1.142(0.867-1.504)	0.344	1.258(0.911-1.736)	0.163	1.160(0.837-1.608)	0.373	1.181(0.871-1.600)	0.284	1.326(1.068-1.647)	0.010*
Education backgrond										
PhD & Master vs. College degree and others	1.743(0.879-3.453)	0.111	1.887(0.919-3.857)	0.083	1.816(0.815-4.044)	0.144	3.108(1.131-8.540)	0.028*	2.399(1.469-3.916)	<0.001*
Bachelor vs. College degree and others	1.208(0.910-1.603)	0.192	1.267(0.906-1.773)	0.167	1.134(0.830-1.551)	0.430	1.417(1.150-1.745)	0.001*	1.378(1.174-1.616)	<0.001*
Professional Title										
Advanced vs. Primary	1.412(0.813-2.452)	0.221	1.050(0.479-2.305)	0.902	2.369(1.368-4.103)	0.002*	0.886(0.498-1.577)	0.681	1.124(0.724-1.745)	0.601
Medium-grade vs. Primary	0.992(0.743-1.323)	0.955	0.618(0.432-0.883)	0.008*	1.060(0.813-1.382)	0.668	1.010(0.759-1.343)	0.946	1.000(0.814-1.227)	0.999
**Years of experience**	1.002(0.996-1.007)	0.563	1.002(0.940-1.069)	0.947	1.043(0.987-1.102)	0.131	1.008(0.986-1.030)	0.474	1.102(1.002-1.023)	0.017*
**Management position (Yes/No)**	1.094(0.810-1.477)	0.489	1.135(0.795-1.620)	0.485	1.088(0.776-1.524)	0.626	1.005(0.777-1.300)	0.970	1.149(0.943-1.400)	0.167
Marital status										
Unmarried vs. married	0.653(0.313-1.361)	0.255	0.807(0.484-1.345)	0.419	0.985(0.616-1.573)	0.948	1.459(1.002-2.115)	0.049*	1.147(0.868-1.517)	0.335
Divorced vs. married	0.937(0.600-1.463)	0.775	1.686(0.576-4.930)	0.340	1.019(0.394-2.636)	0.968	1.364(0.732-2.543)	0.329	1.412(0.818-2.438)	0.216
**Has o child (Yes/No)**	1.152(0.758-1.749)	0.508	0.745(0.467-1.189)	0.217	1.110(0.728-1.693)	0.627	1.048(0.736-1.491)	0.797	0.945(0.725-1.231)	0.674
**Living alone (Yes/No)**	1.098(0.842-1.432)	0.489	1.017(0.747-1.386)	0.914	0.820(0.610-1.103)	0.189	0.798(0.633-1.005)	0.056	0.837(0.697-1.006)	0.058
**A. Whether to treat COVID-19 patients directly during the first big wave (Yes/No)**	/	/	/	/	/	/	/	/	1.010(0.830-1.230)	0.919
**B. Whether to treat COVID-19 patients directly (Yes/No)**	1.370(1.101-1.705)	0.005*	1.409(1.091-1.819)	0.009*	1.495(1.167-1.916)	0.001*	1.324(1.069-1.641)	0.010*	1.065(0.795-1.426)	0.674
A*B	/	/	/	/	/	/	/	/	0.786(0.534-1.156)	0.221
**Total support score**	1.038(0.988-1.090)	0.143	0.988(0.925-1.055)	0.718	0.956(0.902-1.014)	0.132	0.954(0.911-1.000)	0.052	0.974(0.943-1.006)	0.110
**Total score of stress coping strategies**	1.130(1.094-1.167)	<0.001*	1.148(1.108-1.189)	<0.001*	1.144(1.107-1.182)	<0.001*	1.131(1.103-1.160)	<0.001*	1.109(1.089-1.129)	<0.001*

*indicates a statistically significant difference.

### 3.6 Risk factors associated with severe psychological distress

Logistic regression analyses consistently demonstrated that increased social support scores significantly mitigated the risk of severe psychological distress. However, interestingly, a higher aggregate score on stress coping strategies was found to be predictive of a heightened risk of severe psychological distress, hinting at the possibility that some coping strategies might be ineffectual or could potentially exacerbate negative emotional outcomes. The results of logistic regression analysis examining the factors associated with severe psychological distress are presented in **Figure 4** and **Table 4**. A higher total support score was associated with a lower risk of severe psychological distress at T0 (OR, 0.728; 95% CI, 0.666 – 0.796), T1 (OR, 0.771; 95% CI, 0.689 – 0.864), T2 (OR, 0.779; 95% CI, 0.703 – 0.862), T3 (OR, 0.754; 95% CI, 0.704 – 0.808), and T4 (OR, 0.798; 95%CI, 0.764 – 0.833). However, A higher total score of stress coping strategies was associated with an increased risk of severe psychological distress at T0 (OR, 1.245; 95% CI, 1.163 – 1.334), T1 (OR, 1.240; 95% CI, 1.150 – 1.337), T2 (OR, 1.209; 95% CI, 1.142 – 1.280), T3 (OR, 1.183; 95% CI, 1.128 – 1.240) and T4 (OR, 1.216; 95%CI, 1.185 – 1.249).

**Table 4:** Multivariable logistic regression analysis of the factors associated with severe psychological distress.

	T0		T1		T2		T3		T4	
	OR(95%CI)	P Value	OR(95%CI)	P Value	OR(95%CI)	P Value	OR(95%CI)	P Value	OR(95%CI)	P Value
**Age (Years)**	0.996(0.978-1.015)	0.694	1.045(0.860-1.271)	0.656	1.008(0.870-1.169)	0.911	0.961(0.901-1.024)	0.222	0.950(0.893-1.010)	0.102
**Gender (Male/Female)**	0.905(0.471-1.738)	0.764	1.458(0.628-3.384)	0.38	0.922(0.436-1.953)	0.833	0.761(0.386-1.498)	0.429	1.078(0.745-1.561)	0.69
Education backgrond										
PhD & Master vs. College degree and others	1.146(0.215-6.119)	0.873	0.312(0.033-2.960)	0.31	0.621(0.097-3.993)	0.616	1.780(0.207-15.301)	0.599	0.518(0.219-1.222)	0.133
Bachelor vs. College degree and others	1.416(0.660-3.041)	0.372	0.673(0.286-1.586)	0.366	0.780(0.395-1.538)	0.473	1.302(0.816-2.077)	0.268	0.827(0.614-1.115)	0.213
Professional Title										
Advanced vs. Primary	0.864(0.175-4.257)	0.857	2.101(0.242-18.237)	0.501	0.433(0.062-3.041)	0.400	0.688(0.194-2.436)	0.562	0.950(0.450-2.006)	0.893
Medium-grade vs. Primary	0.910(0.443-1.869)	0.796	2.244(0.756-6.654)	0.145	0.795(0.333-1.896)	0.605	0.712(0.399-1.271)	0.251	1.067(0.745-1.528)	0.726
**Years of experience**	0.989(0.930-1.052)	0.72	0.928(0.781-1.103)	0.395	1.001(0.875-1.145)	0.99	1.030(0.989-1.073)	0.15	1.029(0.973-1.090)	0.317
**Management position (Yes/No)**	1.119(0.581-2.156)	0.738	1.877(0.790-4.461)	0.154	1.626(0.783-3.376)	0.193	0.803(0.463-1.392)	0.435	1.264(0.912-1.754)	0.16
Marital status										
Unmarried vs. married	0.477(0.059-3.834)	0.486	0.697(0.192-2.522)	0.582	0.665(0.261-1.698)	0.394	1.106(0.499-2.453)	0.804	0.662(0.407-1.079)	0.098
Divorced vs. married	0.456(0.178-1.215)	0.118	1.595(0.161-15.808)	0.69	1.281(0.179-9.173)	0.805	1.876(0.706-4.984)	0.207	1.027(0.421-2.508)	0.953
**Has o child (Yes/No)**	0.747(0.316-1.768)	0.507	0.747(0.245-2.279)	0.609	0.520(0.299-0.904)	0.020*	1.383(0.660-2.897)	0.391	1.091(0.689-1.727)	0.711
**Living alone (Yes/No)**	1.009(0.529-1.925)	0.977	1.816(0.838-3.935)	0.131	0.751(0.371-1.521)	0.427	0.895(0.548-1.463)	0.658	1.419(1.028-1.959)	0.033
**A. Whether to treat COVID-19 patients directly during the first big wave (Yes/No)**	/	/	/	/	/	/	/	/	0.908(0.637-1.296)	0.596
**B. Whether to treat COVID-19 patients directly (Yes/No)**	0.907(0.537-1.532)	0.714	0.140(0.053-0.374)	<0.001*	1.011(0.559-1.829)	0.97	1.022(0.640-1.633)	0.926	1.008(0.613-1.659)	0.974
A*B	/	/	/	/	/	/	/	/	1.274(0.661-2.456)	0.469
**Total support score**	0.728(0.666-0.796)	<0.001*	0.771(0.689-0.864)	<0.001*	0.779(0.703-0.862)	<0.001*	0.754(0.704-0.808)	<0.001*	0.798(0.764-0.833)	<0.001*
**Total score of stress coping strategies**	1.245(1.163-1.334)	<0.001*	1.240(1.150-1.337)	<0.001*	1.209(1.142-1.280)	<0.001*	1.183(1.128-1.240)	<0.001*	1.216(1.185-1.249)	<0.001*

*indicates a statistically significant difference.

### 3.7 Risk factors associated with PTSD

Logistic regression analyses unveiled that elevated social support scores exerted a protective effect against the development of PTSD. Conversely, a greater aggregate score on stress coping strategies was found to be positively correlated with PTSD, indicating that certain coping mechanisms might be ineffective or potentially detrimental, exacerbating adverse outcomes. The results of logistic regression analysis examining the factors associated with PTSD are presented in **Figure 4** and **Table 5**. A higher total support score was associated with a lower risk of PTSD at T1 (OR, 0.788; 95% CI, 0.722 – 0.860), T2 (OR, 0.822; 95% CI, 0.760 – 0.888), T3 (OR, 0.798; 95% CI, 0.750 – 0.850), and T4 (OR, 0.789; 95%CI, 0.760 – 0.820). However, a higher total score of stress coping strategies was associated with an increased risk of PTSD at T1 (OR, 1.173; 95% CI, 1.110 – 1.240), T2 (OR, 1.245; 95% CI, 1.184 – 1.310), T3 (OR, 1.231; 95% CI, 1.180 – 1.283) and T4 (OR, 1.219; 95%CI, 1.189 – 1.249).

**Table 5:** Multivariable logistic regression analysis of the factors associated with PTSD.

	T1		T2		T3		T4	
	OR(95%CI)	P Value	OR(95%CI)	P Value	OR(95%CI)	P Value	OR(95%CI)	P Value
**Age (Years)**	1.035(0.901-1.190)	0.624	1.035(0.936-1.145)	0.501	1.018(0.967-1.071)	0.5	0.982(0.931-1.035)	0.488
**Gender (Male/Female)**	1.807(1.802-3.018)	0.024*	1.055(0.622-1.789)	0.842	0.869(0.518-1.460)	0.596	1.394(1.051-1.850)	0.021*
Education backgrond								
PhD & Master vs. College degree and others	0.250(0.029-2.132)	0.205	0.808(0.213-3.065)	0.754	0.608(0.074-5.103)	0.644	0.696(0.358-1.350)	0.283
Bachelor vs. College degree and others	1.058(0.549-2.039)	0.866	1.152(0.686-1.934)	0.592	1.110(0.761-1.619)	0.588	0.830(0.646-1.067)	0.147
Professional Title								
Advanced vs. Primary	0.202(0.020-2.006)	0.172	0.908(0.276-2.985)	0.874	1.175(0.460-3.004)	0.736	1.175(0.631-2.188)	0.611
Medium-grade vs. Primary	1.262(0.636-2.505)	0.506	1.136(0.650-1.987)	0.654	1.020(0.637-1.632)	0.935	1.237(0.915-1.673)	0.167
**Years of experience**	0.940(0.831-1.063)	0.321	0.983(0.898-1.077)	0.713	0.987(0.951-1.025)	0.504	1.002(0.955-1.052)	0.933
**Management position (Yes/No)**	1.176(0.619-2.233)	0.621	1.181(0.704-1.982)	0.528	0.753(0.483-1.174)	0.211	1.143(0.864-1.512)	0.35
Marital status								
Unmarried vs. married	0.884(0.303-2.578)	0.821	1.032(0.471-2.259)	0.937	0.602(0.336-1.078)	0.088	1.005(0.659-1.533)	0.98
Divorced vs. married	2.548(0.609-10.660)	0.2	0.900(0.181-4.479)	0.897	1.042(0.439-2.475)	0.926	0.672(0.280-1.616)	0.375
**Has o child (Yes/No)**	2.120(0.840-5.350)	0.112	0.932(0.456-1.905)	0.846	0.674(0.394-1.154)	0.15	1.210(0.807-1.813)	0.356
**Living alone (Yes/No)**	1.520(0.867-2.665)	0.144	1.267(0.785-2.045)	0.333	1.435(1.019-2.021)	0.039*	1.214(0.925-1.594)	0.162
**A. Whether to treat COVID-19 patients directly during the first big wave (Yes/No)**	/	/	/	/	/	/	1.102(0.825-1.472)	0.512
**B. Whether to treat COVID-19 patients directly (Yes/No)**	0.581(0.344-0.983)	0.043	0.893(0.590-1.349)	0.59	0.956(0.657-1.391)	0.815	1.306(0.882-1.934)	0.182
A*B	/	/	/	/	/	/	0.845(0.497-1.436)	0.534
**Total support score**	0.788(0.722-0.860)	<0.001*	0.822(0.760-0.888)	<0.001*	0.798(0.750-0.850)	<0.001*	0.789(0.760-0.820)	<0.001*
**Total score of stress coping strategies**	1.173(1.110-1.240)	<0.001*	1.245(1.184-1.310)	<0.001*	1.231(1.180-1.283)	<0.001*	1.219(1.189-1.249)	<0.001*

*indicates a statistically significant difference.

## 4 Discussion

Our study bridges this gap by longitudinally tracking Chinese nurses’ mental health at five key points over two years. Results show a gradual rise in mental health issues, peaking during COVID-19’s second wave, roughly two years after its onset. Additionally, social support and stress coping strategies emerged as crucial factors influencing nurses’ mental health.

Multiple studies have underscored the diverse mental health challenges nurses faced during the COVID-19 pandemic. Meta-analyses indicate that female healthcare workers, especially nurses, suffered from higher rates of psychological issues than their male and medical counterparts (24–26). A Wuhan-based study during the pandemic’s early stages revealed that a notable proportion of medical professionals experienced varying levels of disturbance: 34.4% mild, 22.4% moderate, and 6.2% severe (27). COVID-19 significantly impacted the psychological wellbeing of frontline hospital staff, increasing their risk of poor mental health outcomes (28). Despite this, long-term studies monitoring nurses’ mental health are scarce.

### 4.1 The trajectory of psychological distress and PTSD among nurses appears to steadily deteriorate over time following the COVID-19 outbreak

Our findings revealed a notable increase in psychological distress and PTSD among nurses during the two-year period following the COVID-19 outbreak. During the COVID-19 pandemic, hospital caregivers were reported experiencing symptoms of anxiety (55%), depression (32%), PTSD (47%) and insomnia (52%) (29). Two to three months later, 54% of healthcare workers exhibited PTSD symptoms, 89% persisted (29).

However, these studies vary in location and time, which may account for the wide variation in their results. Teo et al., found a monthly rise of 1% in stressed HCWs over 6 months, with no significant anxiety increase post-lockdown (30). Similarly, Gundogmus et al.’s research showed that anxiety and depression among HCWs rose with COVID-19 cases, and the pandemic’s duration worsened these mental health issues (31). Marsden and his colleagues tracked anxiety, depression, and PTSD levels in healthcare workers over a year, noting some fluctuations (32). A Japanese survey found improved anxiety and depression scores after six months, but no significant reduction in moderate to severe symptoms (33). These differing results may be due to survey durations or epidemic progression.

### 4.2 During the second big wave of the COVID-19 pandemic, Chinese nurses experienced more pronounced psychological challenges compared to the initial surge that occurred over two years earlier

Our research results showed that during the second surge, the incidence of psychological distress, severe psychological distress, and PTSD among all nurses increased significantly. This trend was observed among both frontline and second-line nurses.Even after one year since the pandemic’s onset, women remained particularly susceptible to emotional distress, with nurses constituting the most affected group (34). A longitudinal study in Canada revealed significant increases in anxiety and depression among nurses, with rates rising by 10% and 15% from pre-pandemic to early pandemic stages. Despite stabilizing two months after the outbreak, psychological burden remained higher than pre-pandemic levels. (17). Likewise, a longitudinal study conducted over six months in Australasia reported the highest incidence of symptoms of depressive and PTSD at 25% and 35%, respectively (15). A study conducted in the United States revealed that levels of anxiety and depression among medical staff were comparable to those observed in the general population (35).

### 4.3 Throughout the COVID-19 pandemic, nurses’ mental health has been worsening, with direct COVID-19 patient contact leading to more severe distress and PTSD

After the relaxation of stress levels at T1, the incidence of severe psychological distress demonstrated a downward trend among-line nurses, whereas no change was observed among first-line nurses. Following the lifting of Wuhan’s lockdown, the incidence of severe psychological distress among first-line nurses was higher than that among second-line nurses at T1, T2, T3 and T4. When the second major wave of the COVID-19 pandemic hit after two years, the severity of psychological distress among first-line nurses surpassed that among second-line nurses. In terms of depression, there was no significant difference observed between first-line and second-line nurses (OR, 1.078; 95% CI, 0.784–1.481) (12). However, both groups reported a noteworthy proportion of participants experiencing symptoms of depression (46.0% for the aiding Wuhan group vs. 49.0% for the controls), anxiety (40.0% vs. 38.0%), and PTSD (61.0% vs. 56.0%) (16).

Our study revealed fluctuations in the incidence of mild-to-moderate stress among both first-line and second-line nurses, with no statistically significant reduction observed by T4. Following the relaxation of stress levels at T1 (China’s Wuhan began to lift its Coronavirus lockdown), the incidence of mild-to-moderate psychological distress in both first-line and second-line remained higher compared to T0. This observation suggests that local lockdowns may have predicted subsequent deterioration in mental health (36). Notably, at T0, the incidence was higher among the second-line nurses compared to the first-line. Frontline workers was reported significantly elevated levels of anxiety (36%) (37). Similarly, Ohue et al. found it was between 20% and 30% (38). Contact with COVID-19 patients emerged as a significant predictor of psychological distress (39, 40). Subgroup analyses conducted by Xiong et al. revealed a higher prevalence of psychological impact among female healthcare workers, frontline workers, nurses, and those working in Wuhan (41).

Bani Issa et al. reported a probable diagnosis of PTSD in 36.2% of nurses (42). Similarly, Bassi et al. found a PTSD diagnosis in 39.8% of nurses and frontline staff being more susceptible (43). In our study, we examined the incidence of PTSD in nurses at four time points (T1, T2, T3, and T4) following the lifting of Wuhan’s lockdown. The incidence consistently increased over time, with higher rates observed among first-line nurses compared to second-line at all time points. However, the difference in incidence between first-line and second-line nurses gradually decreased over time. This trend may be attributed to the ongoing COVID-19 pandemic, as the number of infections and the spread of the epidemic expanded. With the increasing number of patients, anxiety disorders and the intention to resign also heightened (38). Consequently, both first-line and second-line nurses remained at a high risk of contracting COVID-19.

### 4.4 Stress coping strategies exhibit an independent correlation with all grades of psychological distress and PTSD, whereas social support emerges as an independent protective factor mitigating the risk of severe psychological distress and PTSD

Our findings confirmed that the total score of social support independently predicts severe psychological distress and PTSD across all survey time points. With one-point increase in the total social support score, the incidence of severe psychological distress and PTSD decreased by 20.2% to 27.2% and 17.8% to 21.2%, respectively. Notably, family support (95.9%) and management recognition (90.8%) were reported as the most common motivating factors (42). Stricter COVID-19 policies have been associated with poorer mental health outcomes, while strategies aimed at elimination have reduced transmission and deaths at the expense of mental well-being (44). Additionally, research by Muller and his colleagues suggested that social support is associated with lower levels of mental health problems (4). The score of social support is also negatively correlated with depression (45). In the post-epidemic period of COVID-19, nurses continue to face significant physical and psychological risk, highlighting the ongoing need for support to safeguard their well-being (46). Family health plays a significant mediating role between health literacy and mental health (47). It is also essential to pay attention to the family support of nurses’ mental health. Future interventions for nurses’ mental health should focus on both social and family support.

On the other hand, for one-point increased in the total score of stress coping strategies, the incidence of psychological distress, severe psychological distress, and PTSD increase by 10.9% to 14.8%, 18.3% to 24.5%, and 17.3% to 24.5% respectively. Implementing reasonable coping strategies has the potential to improve psychological distress and PTSD caused by the COVID-19 pandemic (48). Notably, emotion-focused strategies show a negative direct and indirect relationship with nurses’ psychological distress through resilience, while problem-focused strategies exhibit a positive direct effect on distress and negative indirect effects through emotion-focused strategies (49). The discrepancy observed between our results and the aforementioned notion could potentially be attributed to the lack of differentiation between active and passive coping strategies in our assessment framework. Among the stress coping strategies (Mini- COPE), younger nurses commonly mentioned venting, instrumental support, a sense of humour, and self-blame. (50). Nurses who often used all strategies, except rejecting unreliable info, had lower psychological distress than those who rarely or never did. Promoting mental health via activities, relaxation, healthy diet, water intake, breaks, social contacts, and emotional expression is key to reducing nurses’ stress and anxiety during COVID-19 (51). It is worth noting that individuals who receive less social support and exhibit negative stress-coping styles are more likely to experience psychological problems (52). The mental health and coping strategies of nurses during the COVID-19 pandemic have significant implications for their ability to provide care and fullfill their responsibilities. Thus, the importance of psychosocial coping methods and support from family, friends, the public, and government cannot be overstated (53). While our study has demonstrated an independent association between stress coping strategies and mental stress as well as PTSD among nurses, it must be acknowledged that the specific linkages between coping strategies and these mental health outcomes have not been fully elucidated within the scope of our research. This limitation originates from the inability of certain coping strategies examined in our study to differentiate between those that exert a beneficial impact and those that are detrimental. Notwithstanding, our findings at least substantiate an independent correlation between coping strategies and nurses’ mental stress and PTSD, implying that the rational formulation of stress coping strategies could potentially contribute to the improvement of nurses’ mental health. Further research, however, is warranted to provide more granular evidence on this subject.

### 4.5 Limitations

Firstly, this longitudinal tracking survey conducted five cross-sectional surveys, all of which were conducted at key time points during the pandemic. To ensure an adequate sample size and genuine responses from participants, no sensitive information of the subjects was collected. This includes information such as IP addresses and contact details, which were not gathered. Consequently, it is impossible to correlate or match the results from the five surveys on an individual basis. However, the researchers distributed the questionnaires using the same approach, requirements, and methods each time to ensure that the participants were as consistent as possible within the same population, thus ensuring the representativeness of the five sampling occasions. Secondly, this study adopts self-report surveys, which may be subject to limitations such as supervisor bias, recall bias, and incomplete data, potentially impacting the survey results. Throughout the entire data collection process, we have endeavored to mitigate these limitations by refining the questionnaire design, expanding the sample collection, incorporating objective indicators where feasible, conducting anonymous surveys (minimizing the collection of personal information), training interviewers, and adjusting the parameters of the “SO JUMP” platform. Thirdly, due to the adoption of snowball sampling in this study, it is not feasible to precisely calculate the number of individuals contacted during the process of requesting recommendations. Consequently, the response rate cannot be determined. Forthly, due to the controversial pros and cons of some coping strategies investigated, such as “seeking support from religious beliefs” and “venting emotions through social media,” we were ultimately unable to distinguish between positive and negative coping strategies. We could only demonstrate that coping strategies are correlated with mental health outcomes, but we cannot determine whether their impact is positive or negative.

## 5 Conclusion

This longitudinal study tracked Chinese nurses over a period of more than two years to assess their mental health. We examined psychological stress and post-traumatic stress disorder (PTSD) at five crucial time points. Our findings reveal a gradual deterioration in nurses’ mental health, including psychological issues, severe psychological issues, and PTSD, with the second major wave exacerbating these effects. The disparity in psychological disorders between frontline and second-line nurses has decreased over time. The study identifies nurses’ stress coping strategies and social support as independent factors influencing their mental well-being. Nursing managers should promptly provide reasonable stress coping strategies and sufficient support to protect the mental health of nurses. Meanwhile, in the response to future public health emergencies, reasonable stress coping strategies and sufficient support for nurses should also be considered.

## Data Availability

The original contributions presented in the study are included in the article/**Supplementary Material**. Further inquiries can be directed to the corresponding author.

## References

[1] Alshammari MA Alshammari TK. COVID-19: A new challenge for mental health and policymaking recommendations. J Infect Public Health. (2021) 14:1065–8. doi: 10.1016/j.jiph.2021.05.020 PMC8188780 34174536

[2] Cui J Lu J Weng Y Yi GY He W. COVID-19 impact on mental health. BMC Med Res Methodol. (2022) 22:15. doi: 10.1186/s12874-021-01411-w 35026998 PMC8758244

[3] Zhang X Zhou Y Fan C Huang X Long L Yu S. Visualization and bibliometric analysis of occupational exposure among nurses in Asia. Heliyon. (2023) 9. doi: 10.1016/j.heliyon.2023.e21289 PMC10598530 37885731

[4] Muller AE Hafstad EV Himmels JPW Smedslund G Flottorp S Stensland SO. The mental health impact of the covid-19 pandemic on healthcare workers, and interventions to help them: A rapid systematic review. Psychiatry Res. (2020) 293:113441. doi: 10.1016/j.psychres.2020.113441 32898840 PMC7462563

[5] Vindegaard N Benros ME. COVID-19 pandemic and mental health consequences: Systematic review of the current evidence. Brain Behav Immun. (2020) 89:531–42. doi: 10.1016/j.bbi.2020.05.048 PMC7260522 32485289

[6] AlAteeq DA Aljhani S Althiyabi I Majzoub S. Mental health among healthcare providers during coronavirus disease (COVID-19) outbreak in Saudi Arabia. J Infect Public Health. (2020) 13:1432–7. doi: 10.1016/j.jiph.2020.08.013 PMC7834809 32933881

[7] Dragioti E Tsartsalis D Mentis M Mantzoukas S Gouva M. Impact of the COVID-19 pandemic on the mental health of hospital staff: An umbrella review of 44 meta-analyses. Int J Nurs Stud. (2022) 131:104272. doi: 10.1016/j.ijnurstu.2022.104272 35576637 PMC9045868

[8] K, N. M Parashar N Kumar CRS Verma V Rao S Y S. Prevalence and severity of secondary traumatic stress and optimism in Indian health care professionals during COVID-19 lockdown. PloS One. (2021) 16:e0257429. doi: 10.1371/journal.pone.0257429 34582481 PMC8478227

[9] Yunitri N Chu H Kang XL Jen HJ Pien LC Tsai HT. Global prevalence and associated risk factors of posttraumatic stress disorder during COVID-19 pandemic: A meta-analysis. Int J Nurs Stud. (2022) 126:104136. doi: 10.1016/j.ijnurstu.2021.104136 34856503 PMC8585564

[10] Isik M Kirli U Ozdemir PG. The mental health of healthcare professionals during the COVID-19 pandemic. Turk Psikiyatri Derg. (2021) 32:225–34. doi: 10.5080/u25827 34964096

[11] Xu L You D Li C Zhang X Yang R Kang C. Two-stage mental health survey of first-line medical staff after ending COVID-19 epidemic assistance and isolation. Eur Arch Psychiatry Clin Neurosci. (2022) 272:81–93. doi: 10.1007/s00406-021-01239-x 34008059 PMC8130787

[12] Zheng R Zhou Y Fu Y Xiang Q Cheng F Chen H. Prevalence and associated factors of depression and anxiety among nurses during the outbreak of COVID-19 in China: A cross-sectional study. Int J Nurs Stud. (2021) 114:103809. doi: 10.1016/j.ijnurstu.2020.103809 33207297 PMC7583612

[13] Liu Y Long Y Cheng Y Guo Q Yang L Lin Y. Psychological impact of the COVID-19 outbreak on nurses in China: A nationwide survey during the outbreak. Front Psychiatry. (2020) 11:598712. doi: 10.3389/fpsyt.2020.598712 33362609 PMC7759517

[14] Couper K Murrells T Sanders J Anderson JE Blake H Kelly D. The impact of COVID-19 on the wellbeing of the UK nursing and midwifery workforce during the first pandemic wave: A longitudinal survey study. Int J Nurs Stud. (2022) 127:104155. doi: 10.1016/j.ijnurstu.2021.104155 35093740 PMC8673915

[15] Ng I Barson E Fisher C Segal R Williams DL Krieser RB. A longitudinal study of the psychological impact of the COVID-19 pandemic on frontline perioperative healthcare staff in an Australian tertiary public hospital. Australas Psychiatry. (2022) 30:212–22. doi: 10.1177/10398562221077887 PMC8927895 35285740

[16] Zhang R Lai J Wang Y Huang J Hu S Wang H. Mental health outcome and resilience among aiding Wuhan nurses: One year after the COVID-19 outbreak in China. J Affect Disord. (2022) 297:348–52. doi: 10.1016/j.jad.2021.10.050 PMC8564215 34710499

[17] Havaei F Smith P Oudyk J Potter GG. The impact of the COVID-19 pandemic on mental health of nurses in British Columbia, Canada using trends analysis across three time points. Ann Epidemiol. (2021) 62:7–12. doi: 10.1016/j.annepidem.2021.05.004 34052436 PMC9536865

[18] Kendall C Kerr LR Gondim RC Werneck GL Macena RH Pontes MK. An empirical comparison of respondent-driven sampling, time location sampling, and snowball sampling for behavioral surveillance in men who have sex with men, Fortaleza, Brazil. AIDS Behav. (2008) 12:S97–104. doi: 10.1007/s10461-008-9390-4 18389357

[19] NCNA. At zero o 'clock on April 8, the exit control of Wuhan was officially lifted (2020). Available online at: http://www.gov.cn/xinwen/2020-04/08/content_5500100.htm (Accessed on March 12, 2024).

[20] Bridges KW Goldberg DP. The validation of the GHQ-28 and the use of the MMSE in neurological in-patients. Br J Psychiatry. (1986) 148:548–53. doi: 10.1192/bjp.148.5.548 3779225

[21] Goldberg DP Hillier VF. A scaled version of the General Health Questionnaire. psychol Med. (1979) 9:139–45. doi: 10.1017/s0033291700021644 424481

[22] Liu XC Ma DD Liu Q. Development of the post-traumatic stress disorder self-rating scale and its reliability and validity. Chin J Behav Med Sci. (1998) 7:93–6.

[23] Armstrong RA. When to use the Bonferroni correction. Ophthalmic Physiol Opt. (2014) 34:502–8. doi: 10.1111/opo.12131 24697967

[24] Ching SM Ng KY Lee KW Yee A Lim PY Ranita H. Psychological distress among healthcare providers during COVID-19 in Asia: Systematic review and meta-analysis. PloS One. (2021) 16:e0257983. doi: 10.1371/journal.pone.0257983 34648526 PMC8516240

[25] Luo M Guo L Yu M Jiang W Wang H. The psychological and mental impact of coronavirus disease 2019 (COVID-19) on medical staff and general public – A systematic review and meta-analysis. Psychiatry Res. (2020) 291. doi: 10.1016/j.psychres.2020.113190 PMC7276119 32563745

[26] Pappa S Ntella V Giannakas T Giannakoulis VG Papoutsi E Katsaounou P. Prevalence of depression, anxiety, and insomnia among healthcare workers during the COVID-19 pandemic: A systematic review and meta-analysis. Brain Behav Immun. (2020) 88:901–7. doi: 10.1016/j.bbi.2020.05.026 PMC7206431 32437915

[27] Kang L Ma S Chen M Yang J Wang Y Li R. Impact on mental health and perceptions of psychological care among medical and nursing staff in Wuhan during the 2019 novel coronavirus disease outbreak: A cross-sectional study. Brain Behav Immun. (2020) 87:11–7. doi: 10.1016/j.bbi.2020.03.028 PMC7118532 32240764

[28] De Kock JH Latham HA Leslie SJ Grindle M Munoz SA Ellis L. A rapid review of the impact of COVID-19 on the mental health of healthcare workers: implications for supporting psychological well-being. BMC Public Health. (2021) 21:104. doi: 10.1186/s12889-020-10070-3 33422039 PMC7794640

[29] Mennicken B Petit G Yombi JC Belkhir L Deschietere G Germeau N. Psychological distress among hospital caregivers during and after the first wave of COVID-19: Individual factors involved in the severity of symptoms expression. Psychiatry Res Commun. (2022) 2:100037. doi: 10.1016/j.psycom.2022.100037 35496465 PMC9040471

[30] Teo I Chay J Cheung YB Sung SC Tewani KG Yeo LF. Healthcare worker stress, anxiety and burnout during the COVID-19 pandemic in Singapore: A 6-month multi-centre prospective study. PloS One. (2021) 16:e0258866. doi: 10.1371/journal.pone.0258866 34679110 PMC8535445

[31] Gundogmus I Bolu A Unsal C Alma L Gundogmus PD Takmaz T. Impact of the first, second and third peak of the COVID-19 pandemic on anxiety, depression and stress symptoms of healthcare workers. Bratislava Med J. (2022) 123:833–9. doi: 10.4149/bll_2022_133 36254642

[32] Marsden KM Robertson IK Porter J. Stressors, manifestations and course of COVID-19 related distress among public sector nurses and midwives during the COVID-19 pandemic first year in Tasmania, Australia. PloS One. (2022) 17:e0271824. doi: 10.1371/journal.pone.0271824 35944016 PMC9362919

[33] Awano N Oyama N Akiyama K Inomata M Kuse N Tone M. Comparison of Mental Health among Japanese Healthcare Workers at Two Points during the COVID-19 Pandemic. J Nippon Med Sch. (2022) 89:328–36. doi: 10.1272/jnms.JNMS.2022_89-308 35768270

[34] Garcia-Fernandez L Romero-Ferreiro V Rodriguez V Alvarez-Mon MA Lahera G Rodriguez-Jimenez R. What about mental health after one year of COVID-19 pandemic? A comparison with the initial peak. J Psychiatr Res. (2022) 153:104–8. doi: 10.1016/j.jpsychires.2022.07.010 PMC9254135 35810599

[35] Schechter-Finkelstein T Plenert E La Rosa J McLean J Chiang KY Krueger J. Pediatric hematology/oncology healthcare professional emotional health during COVID-19. Cancer Med. (2021) 10:7144–51. doi: 10.1002/cam4.4253 PMC8525130 34467652

[36] Pierce M McManus S Hope H Hotopf M Ford T Hatch SL. Mental health responses to the COVID-19 pandemic: a latent class trajectory analysis using longitudinal UK data. Lancet Psychiatry. (2021) 8:610–9. doi: 10.1016/S2215-0366(21)00151-6 PMC9764381 33965057

[37] Saddik B Elbarazi I Temsah MH Saheb Sharif-Askari F Kheder W Hussein A. Psychological distress and anxiety levels among health care workers at the height of the COVID-19 pandemic in the United Arab Emirates. Int J Public Health. (2021) 66:1604369. doi: 10.3389/ijph.2021.1604369 34840553 PMC8615074

[38] Ohue T Togo E Ohue Y Mitoku K. Mental health of nurses involved with COVID-19 patients in Japan, intention to resign, and influencing factors. Med (Baltimore). (2021) 100:e26828. doi: 10.1097/MD.0000000000026828 PMC8341249 34397847

[39] Dziedzic B Kobos E Sienkiewicz Z Idzik A. Mental health of nurses during the fourth wave of the COVID-19 pandemic in Poland. Int J Environ Res Public Health. (2022) 19. doi: 10.3390/ijerph19031785 PMC8835120 35162808

[40] Gorini A Fiabane E Sommaruga M Barbieri S Sottotetti F La Rovere MT. Mental health and risk perception among Italian healthcare workers during the second month of the Covid-19 pandemic. Arch Psychiatr Nurs. (2020) 34:537–44. doi: 10.1016/j.apnu.2020.10.007 PMC7577253 33280678

[41] Xiong N Fritzsche K Pan Y Lohlein J Leonhart R. The psychological impact of COVID-19 on Chinese healthcare workers: a systematic review and meta-analysis. Soc Psychiatry Psychiatr Epidemiol. (2022) 57:1515–29. doi: 10.1007/s00127-022-02264-4 PMC8943357 35325261

[42] Bani Issa W Al Nusair H AlTamimi A Rababa M Saqan R Hijazi H. Posttraumatic stress disorders and influencing factors during the COVID-19 pandemic: A cross-sectional study of frontline nurses. Int Nurs Rev. (2021) 69:285–93. doi: 10.1111/inr.12734 34878183

[43] Bassi M Negri L Delle Fave A Accardi R. The relationship between post-traumatic stress and positive mental health symptoms among health workers during COVID-19 pandemic in Lombardy, Italy. J Affect Disord. (2021) 280:1–6. doi: 10.1016/j.jad.2020.11.065 PMC9188831 33220632

[44] Aknin LB Andretti B Goldszmidt R Helliwell JF Petherick A De Neve JE. Policy stringency and mental health during the COVID-19 pandemic: a longitudinal analysis of data from 15 countries. Lancet Public Health. (2022) 7:e417–26. doi: 10.1016/S2468-2667(22)00060-3 PMC9023007 35461592

[45] Fang XH Wu L Lu LS Kan XH Wang H Xiong YJ. Mental health problems and social supports in the COVID-19 healthcare workers: a Chinese explanatory study. BMC Psychiatry. (2021) 21:34. doi: 10.1186/s12888-020-02998-y 33435867 PMC7802988

[46] Zhang M-R Huang H-G Chen H-X Deng Y-F. Factors associated with poor mental health outcomes in nurses in COVID-19-designated hospitals in the postepidemic period in Guangdong Province: a cross-sectional study. BMJ Open. (2022) 12. doi: 10.1136/bmjopen-2022-061116 PMC9297002 35851024

[47] Wang D Sun X He F Liu C Wu Y. The mediating effect of family health on the relationship between health literacy and mental health: A national cross-sectional survey in China. Int J Soc Psychiatry. (2023) 69:1490–500. doi: 10.1177/00207640231166628 37095729

[48] Finstad GL Giorgi G Lulli LG Pandolfi C Foti G León-Perez JM. Resilience, coping strategies and posttraumatic growth in the workplace following COVID-19: A narrative review on the positive aspects of trauma. Int J Environ Res Public Health. (2021) 18. doi: 10.3390/ijerph18189453 PMC8468098 34574378

[49] Lorente L Vera M Peiro T. Nurses stressors and psychological distress during the COVID-19 pandemic: The mediating role of coping and resilience. J Adv Nurs. (2021) 77:1335–44. doi: 10.1111/jan.14695 PMC7753515 33210768

[50] Sierakowska M Doroszkiewicz H. Stress coping strategies used by nurses during the COVID-19 pandemic. PeerJ. (2022) 10. doi: 10.7717/peerj.13288 PMC9070319 35529493

[51] Pinho L Correia T Sampaio F Sequeira C Teixeira L Lopes M. The use of mental health promotion strategies by nurses to reduce anxiety, stress, and depression during the COVID-19 outbreak: A prospective cohort study. Environ Res. (2021) 195:110828. doi: 10.1016/j.envres.2021.110828 33548294 PMC7857980

[52] Wu Q Li D Yan M Li Y. Mental health status of medical staff in Xinjiang Province of China based on the normalisation of COVID-19 epidemic prevention and control. Int J Disaster Risk Reduct. (2022) 74:102928. doi: 10.1016/j.ijdrr.2022.102928 35368428 PMC8958729

[53] Maideen AA Idris DR Lupat A Chung YF Haji-Badarudin HS Suhai HK. Nurses' mental health and coping strategies throughout COVID -19 outbreak: A nationwide qualitative study. Int J Ment Health Nurs. (2022) 31:1213–27. doi: 10.1111/inm.13031 PMC9349883 35714038

