# Supplementary material for: Trends and determinants of nurses’ mental health following the COVID-19 pandemic in China: a longitudinal, comparative study over a two-year period
Source: Front Psychiatry. 2024 Nov 7;15:1480969. doi: 10.3389/fpsyt.2024.1480969 (PMC11579488; doi:10.3389/fpsyt.2024.1480969)
Supplement: Supplementary file 1 [file Table1.docx]

|  | **χ2** | **P Value** | **Test level** |
| --- | --- | --- | --- |
| T0 First-/Second-line | 6.069 | 0.014* | 0.05 |
| T1 First-/Second-line | 8.873 | 0.003* |  |
| T2 First-/Second-line | 16.836 | 0.001* |  |
| T3 First-/Second-line | 18.354 | 0.001* |  |
| T4 First-/Second-line | 0.015 | 0.903 |  |
| **T0-T1-T2-T3-T4（All nurses）** | 350.187 | <0.001* | 0.05 |
| T0-T1 | 100.592 | <0.001* | 0.005 |
| T0-T2 | 91.816 | <0.001* |  |
| T0-T3 | 119.233 | <0.001* |  |
| T0-T4 | 344.909 | <0.001* |  |
| T1-T2 | 0.518 | 0.472 |  |
| T1-T3 | 0.117 | 0.733 |  |
| T1-T4 | 34.581 | <0.001* |  |
| T2-T3 | 0.211 | 0.646 |  |
| T2-T4 | 49.594 | <0.001* |  |
| T3-T4 | 56.430 | <0.001* |  |
| **T0-T1-T2-T3-T4（First-line）** | 187.628 | <0.001* | 0.05 |
| T0-T1 | 94.950 | <0.001* | 0.005 |
| T0-T2 | 95.722 | <0.001* |  |
| T0-T3 | 111.824 | <0.001* |  |
| T0-T4 | 151.571 | <0.001* |  |
| T1-T2 | 0.413 | 0.521 |  |
| T1-T3 | 0.758 | 0.384 |  |
| T1-T4 | 3.926 | 0.048 |  |
| T2-T3 | 0.030 | 0.863 |  |
| T2-T4 | 0.338 | 0.247 |  |
| T3-T4 | 1.093 | 0.296 |  |
| **T0-T1-T2-T3-T4（Second-line）** | 211.375 | <0.001* | 0.05 |
| T0-T1 | 19.601 | <0.001* | 0.005 |
| T0-T2 | 19.603 | <0.001* |  |
| T0-T3 | 32.073 | <0.001* |  |
| T0-T4 | 162.655 | <0.001* |  |
| T1-T2 | 0.180 | 0.671 |  |
| T1-T3 | 0.082 | 0.775 |  |
| T1-T4 | 39.609 | <0.001* |  |
| T2-T3 | 0.730 | 0.393 |  |
| T2-T4 | 63.281 | <0.001* |  |
| T3-T4 | 69.594 | <0.001* |  |

* indicates a statistically significant difference.

**Supplementary Material 1**: Chi-square test between the incidence of psychological distress in different periods and different categories of nurses.
