# Supplementary material for: Trends and determinants of nurses’ mental health following the COVID-19 pandemic in China: a longitudinal, comparative study over a two-year period
Source: Front Psychiatry. 2024 Nov 7;15:1480969. doi: 10.3389/fpsyt.2024.1480969 (PMC11579488; doi:10.3389/fpsyt.2024.1480969)
Supplement: Supplementary file 2 [file Table2.docx]

|  | **χ2** | **P Value** | **Test level** |
| --- | --- | --- | --- |
| T0 First-/Second-line | 0.079 | 0.779 | 0.05 |
| T1 First-/Second-line | 27.342 | <0.001* |  |
| T2 First-/Second-line | 0.379 | 0.538 |  |
| T3 First-/Second-line | 0.278 | 0.598 |  |
| T4 First-/Second-line | 7.574 | 0.006* |  |
| **T0-T1-T2-T3-T4（All nurses）** | 61.369 | <0.001 | 0.05 |
| T0-T1 | 3.036 | 0.081 | 0.005 |
| T0-T2 | 0.445 | 0.505 |  |
| T0-T3 | 1.165 | 0.280 |  |
| T0-T4 | 20.316 | <0.001* |  |
| T1-T2 | 1.176 | 0.278 |  |
| T1-T3 | 7.772 | 0.005 |  |
| T1-T4 | 33.303 | <0.001* |  |
| T2-T3 | 3.024 | 0.082 |  |
| T2-T4 | 24.813 | <0.001* |  |
| T3-T4 | 14.676 | <0.001* |  |
| **T0-T1-T2-T3-T4（First-line）** | 70.371 | <0.001* | 0.05 |
| T0-T1 | 18.106 | <0.001* | 0.005 |
| T0-T2 | 0.007 | 0.993 |  |
| T0-T3 | 0.221 | 0.638 |  |
| T0-T4 | 19.924 | <0.001* |  |
| T1-T2 | 17.454 | <0.001* |  |
| T1-T3 | 20.917 | <0.001* |  |
| T1-T4 | 58.291 | <0.001* |  |
| T2-T3 | 0.114 | 0.735 |  |
| T2-T4 | 13.840 | <0.001* |  |
| T3-T4 | 13.875 | <0.001* |  |
| **T0-T1-T2-T3-T4（Second-line）** | 20.422 | <0.001* | 0.05 |
| T0-T1 | 1.103 | 0.294 | 0.005 |
| T0-T2 | 0.817 | 0.366 |  |
| T0-T3 | 0.694 | 0.405 |  |
| T0-T4 | 7.161 | 0.007 |  |
| T1-T2 | 3.735 | 0.053 |  |
| T1-T3 | 0.162 | 0.688 |  |
| T1-T4 | 1.527 | 0.217 |  |
| T2-T3 | 3.493 | 0.062 |  |
| T2-T4 | 14.470 | <0.001* |  |
| T3-T4 | 5.437 | 0.020 |  |

* indicates a statistically significant difference.

**Supplementary Material 2**: Chi-square test between the incidence of severe psychological distress in different periods and different categories of nurses.
