# Supplementary material for: Trends and determinants of nurses’ mental health following the COVID-19 pandemic in China: a longitudinal, comparative study over a two-year period
Source: Front Psychiatry. 2024 Nov 7;15:1480969. doi: 10.3389/fpsyt.2024.1480969 (PMC11579488; doi:10.3389/fpsyt.2024.1480969)
Supplement: Supplementary file 3 [file Table3.docx]

|  | **χ2** | **P Value** | **Test level** |
| --- | --- | --- | --- |
| T1 First-/Second-line | 4.742 | 0.029* | 0.05 |
| T2 First-/Second-line | 0.234 | 0.629 |  |
| T3 First-/Second-line | 0.029 | 0.866 |  |
| T4 First-/Second-line | 12.919 | <0.001* |  |
| **T1-T2-T3-T4（All nurses）** | 47.053 | <0.001* | 0.05 |
| T1-T2 | 7.862 | 0.005* | 0.0083 |
| T1-T3 | 4.847 | 0.028 |  |
| T1-T4 | 34.517 | <0.001* |  |
| T2-T3 | 0.769 | 0.381 |  |
| T2-T4 | 9.058 | 0.003* |  |
| T3-T4 | 20.718 | <0.001* |  |
| **T1-T2-T3-T4（First-line）** | 51.790 | <0.001* |  |
| T1-T2 | 10.268 | 0.001* | 0.0083 |
| T1-T3 | 5.939 | 0.015 |  |
| T1-T4 | 44.609 | <0.001* |  |
| T2-T3 | 0.780 | 0.377 |  |
| T2-T4 | 9.794 | 0.002* |  |
| T3-T4 | 18.835 | <0.001* |  |
| **T1-T2-T3-T4（Second-line）** | 13.138 | 0.004* | 0.05 |
| T1-T2 | 0.612 | 0.434 | 0.0083 |
| T1-T3 | 0.223 | 0.637 |  |
| T1-T4 | 6.166 | 0.013 |  |
| T2-T3 | 0.191 | 0.662 |  |
| T2-T4 | 3.721 | 0.054 |  |
| T3-T4 | 8.235 | 0.004* |  |

* indicates a statistically significant difference.

**Supplementary Material 3**: Chi-square test between the incidence of PTSD in different periods and different categories of nurses.
